# The Hearing Aid Effect in the 2020s: Where Do We Stand?

**DOI:** 10.7759/cureus.38302

**Published:** 2023-04-29

**Authors:** Abdullah Sindi, Kamal Hanbazazah, Malak M Alamoudi, Ahd Al-Harbi, Mohammed Aljuhani, Faisal Zawawi

**Affiliations:** 1 Otolaryngology-Head and Neck Surgery, King Abdulaziz University Hospital, Jeddah, SAU; 2 Otolaryngology-Head and Neck Surgery, King Fahd Armed Forces Hospital, Jeddah, SAU; 3 Emergency Medicine, King Abdulaziz University Hospital, Jeddah, SAU; 4 Faculty of Medicine, King Abdulaziz University, Jeddah, SAU; 5 Otolaryngology-Head and Neck Surgery, King Abdulaziz University, Jeddah, SAU

**Keywords:** stigma in health care, baha, cochlear implant (ci) surgery, pediatric hearing loss, hearing aid

## Abstract

Introduction

The ‘hearing aid effect’ is a negative perception toward individuals using hearing assistive devices (HADs), which is a primary reason for parents and children refusing to use them. We aimed to determine the current perception toward individuals using HADs and the associated factors.

Methods

A 30-item photo-based survey was conducted to analyze the participants’ perception toward individuals using HADs as compared to healthy (H) individuals and individuals with disabilities (D). The survey was validated with an intrarater reliability of 86%. A cross-sectional study was conducted by approaching individuals who visited one of the largest shopping centers in a metropolitan city to participate in the survey. Demographic information, including age, gender, and educational background, was collected.

Results

A total of 517 participants completed the survey. Nearly two-thirds of the participants (59.7%) did not consider individuals using HADs as those who needed assistance as compared to H individuals. Interestingly, Generation X and Z participants had a significantly better perception toward individuals using HADs (63.1% and 59%, respectively) as compared to participants of the Baby Boomers generation (54.3%). The majority of participants who considered HD use a handicap compared to healthy individuals (79.9%) did not have a family member that used a HAD.

Conclusion

The stigma of wearing a HAD is significantly reducing with time, and the younger generations are not considering it as a disability. This is an important point that can be highlighted while counseling parents and young adults who are candidates for HAD use.

## Introduction

Hearing loss is a chronic condition that impairs the patient’s ability to communicate. It can occur in children too, causing speech delays. It also has social and emotional effects, causing exclusion from conversations, loneliness, isolation, and frustration. Hearing loss affects not only those with hearing impairment but also those with whom they communicate [1]. A total of 466 million people have disabling hearing loss, and by 2050, one in every 10 individuals could have disabling hearing loss [2-6]. Hearing assistive devices (HADs) are a valuable solution to hearing loss and communication issues; however, only one-third of older United States adults with hearing loss use HADs [7].

The prevalence of hearing loss in Saudi Arabia is approximately 13% in children [8] and 17.35% in adults [9]. Many social and environmental factors contribute to the low proportions of HAD acceptance, including the degree of hearing impairment, cost, access to health care providers, stigma, and the ‘hearing aid effect’ in individuals using HADs [10].

The hearing aid effect has been described in the literature as a negative perception and attitude toward hearing aid users. These perceptions include a reduced sense of capability, handicap, and need for assistance [11,12]. These social perceptions of people should be seriously taken into account, as they can impact HAD acceptance and use in the hearing-disabled community [13-15]. Therefore, the primary purpose of our study is to identify how people of various age groups and educational levels view those who use HADs in society. To our knowledge, no such study has been conducted in Saudi Arabia.

## Materials and methods

Study design and participants

This study was approved by the Institutional Review Board of King Abdulaziz University Faculty of Medicine (Protocol # 61-20). This observational, descriptive, cross-sectional study included the general public visiting one of the largest shopping centers in a metropolitan city of Saudi Arabia over two days (January 30 and 31, 2020). Written informed consent was taken from participants and their parents when participants were under the age of 18.

Data collection methods

A 30-item photo-based survey was created out of commercially available photos comparing individuals using HADs with healthy (H) individuals and individuals with physical disabilities (PDs); these included individuals on wheelchairs, crutches, etc. The questionnaire had three main categories: each category had 10 pictures with a question asking the participant to choose which of the two photos required assistance or special care. The first category compared individuals with PDs and H individuals. The second category compared individuals with PDs and HADs. The third category compared individuals with HADs and H individuals. The 30 questions were not in order and were randomly listed. We also recorded the participants’ demographics including age, which was sub-divided into seven age groups: 5-10, 11-18, 19-25, 26-35, 36-55, 56-75, and >75 years, sex, and education level, which was subdivided into elementary, intermediate, secondary, higher education, read and write, and illiterate. Last, we enquired if any participant had any family member who was using a HAD.

The survey first went through a three-step validation process and pilot study to determine the intra-rater reliability resulting in an intra-rater reliability of 86%.

Examples of the description of the pictures and activities in each category were as follows:

Category 1: An individual with PD walking with a crutch vs. an H individual walking.

Category 2: A child with PD playing the guitar vs. a child using a HAD playing the guitar.

Category 3: A child using a HAD coloring vs. an H child coloring.

The results/observations of the survey were recorded on the pretested semi-structured proforma.

Statistical analysis

All data were analyzed using IBM SPSS Statistics for Windows, version 22.0 (IBM Corp., Armonk, NY). The data were presented as frequency and percentage and were summarized. The chi-square test was used to analyze categorical data. A p-value ≤0.05 was considered statistically significant.

## Results

A total of 627 visitors participated in our survey, of which 517 completed the survey, with a completion rate of 82.46%.

Of the 517 participants, 244 (47.2%) were men and 273 (52.8%) were women (Table 1). The participants were subdivided into seven age groups. Of these, 29.2% were in the 26-35-year age group and 24.6% in the 19-25-year age group. Of the participants, 65.6% had completed higher education and 22.6% had completed secondary school.

**Table 1:** Demographics of the participants

Sex		
Frequency		Percentage
Male	244	47.2
Female	273	52.8
Total	517	100

Age		
Frequency		Percentage
5-10 years	12	2.3
11-18 years	57	11.0
19-25 years	127	24.6
26-35 years	151	29.2
36-55 years	122	23.6
56-75 years	47	9.1
>76 years	1	.2
Total	517	100

Education level		
Frequency		Percentage
Elementary	25	4.8
Intermediate	31	6.0
Secondary	117	22.6
Higher education	339	65.6
Read and write	1	.2
Illiterate	4	.8
Total	517	100

The Category 1 (H vs. PD) comparison showed that 84.6% of the participants responded that the individual with PD in the picture required special care/assistance.

The Category 2 (HAD vs. PD) comparison showed that 79.1% of the participants agreed that the individual using a HAD did not require assistance/special care compared with the individual with PD. Further, 82% of the participants who chose the individual with PD in the first category were consistent and chose the same in the second category.

The Category 3 (HAD vs. H) comparison showed that nearly two-thirds of the participants (59.7%) did not consider the individual using a HAD as a person who requires assistance/special care (p <0.0001).

Men considered an individual using a HAD as a person with a disability (37.8%) to a lesser extent than women (42.5%; p <0.001).

The 36-55-year age group had a better perception of the individual using a HAD and did not consider one as a person with a disability but as a independent person (63.1%), followed by the 19-25-year age group (61.1%) (Table 2).

**Table 2:** Proportion of individuals needing help according to the participants of this study This table highlights the various compared groups and their responses to the questions.

Healthy	1.8%
Physically disabled	84.6%
Neither	13.6%
Total	100%

Hearing aid	9.9%
Physically disabled	79.1%
Neither	11.1%
Total	100%

Hearing aid	40.3%
Healthy	12.3%
Neither	47.4%
Total	100%

Hearing aid	40.3%
No hearing aid (healthy + neither)	59.7%
Total	100%

Participants with a higher education considered an individual using a HAD as self-dependent and not requiring help to a greater extent (61.8%) than participants with lesser education, i.e., with high school or intermediate school education (55.5% and 56%, respectively; p <0.0001).

Of the respondents who considered an individual using a HAD as a person who requires assistance/special care, 79.9% did not have a family member with a HAD.

## Discussion

The aim of this study was to identify the perception of our population toward their peers with HADs when compared to healthy individuals and PD individuals. Did they consider individuals using a HAD as disabled individuals and as those who need help or assistance?

The study results showed that most of our population (59.7%) considered individuals with HADs as independent and not requiring help when compared to healthy individuals, which is consistent with the trend of the reducing hearing aid effect since it was first described in 1977. The effect has been significantly decreasing since then [9], which could be related to the enhancements in the designs of HADs since 1985 [16]. Wireless earbuds, such as those manufactured by different technological companies, may have played a role in diminishing the hearing aid effect [17]. These are likely related to the desensitization of the community of persons wearing a hearing aid or music/phone-related earbuds and speakers. In contrast, Strange et al. found a significant hearing aid effect within the indigenous Australian adolescent population [18].

With regard to age, the perception of the 36-55-year age group was more positive toward individuals using HADs. In contrast, the 11-18-year age group had the most negative attitude toward individuals using HADs, consistent with the findings of Haley and Hood (1986) [19] and Silverman and Klees (1989) [20] who evaluated the perception of young adolescents toward peers using HADs. Likewise, the 56-75-year age group in our study showed a similar impression toward individuals with HADs, which was similar to the findings of McKee et al. [21], who thought that HAD stigma is a significant barrier to its adoption. In addition, Wheeler and Tharpe (2020) [22] and Blood et al. (1978) [23] studied children in the age group of 6-11 years and in elementary school and found that peers who wore hearing aids were depicted negatively. Similarly, our pediatric population had a better perception of individuals using HADs, with 40.8% believing that individuals using HADs needed help or assistance. However, more than half (60%) believed the opposite.

With regard to education, the higher the degree of education of the participant, the lower the hearing aid effect. Participants with higher degrees of education had a better perception of individuals using HADs (61.8%) than participants having secondary school (55.9%) and elementary school (54.8%) education. Few studies have examined the correlation between education and perception toward individuals using HADs. Cox et al. [24] found that college-educated teachers rated individuals using HADs lower on achievement.

McKee et al. found that male participants were less concerned about HAD use than their female counterparts [21]. Moreover, Doggett et al. reported that older women were more likely to consider their peers using HADs as less intelligent, friendly, and confident [12]. In contrast, we did not find any difference between the males and females in regards to their perception toward HAD (62.2% and 57.5%, respectively, reported that HAD were as independent as their healthy counterparts).

This study has a few limitations. Different hearing aid types and designs have not been discussed in detail in our study and the photos used in the study are commercial and publicly available and not designed for the sole purpose of this study. Future studies could compare the use of hearing assistive devices to other common assistive tools (e.g. eyeglasses).

## Conclusions

In conclusion, we found that the hearing aid effect among our population was less than expected, with two-thirds of our participants considering individuals using HADs as independent and not requiring help or assistance in their daily activities. Some differences were noted among sex, age groups, and education levels in the perception toward individuals using HADs. Our data reflect that public health awareness campaigns to clarify misconceptions about hearing loss, and HADs will help in demolishing the stigma and improving the population’s view toward individuals using HADs.
